# Frequency and Clinical Predictors of Cutaneous Manifestations in Patients With Chronic Liver Disease

**DOI:** 10.7759/cureus.111168

**Published:** 2026-06-19

**Authors:** Filza Ajmal, Hamza Ajmal, Dur E Nayab, Samra Tariq, Jawad Ahmad, Ubaid Ur Rahman, Hajra Iftikhar, Zubair Ahmad, Tamanna Nazir, Hanif Ullah Hanfi

**Affiliations:** 1 Dermatology, Hayatabad Medical Complex Medical Teaching Institution, Peshawar, PAK; 2 Medicine, Khyber Medical College, Peshawar, PAK; 3 Internal Medicine, Lady Reading Hospital, Peshawar, PAK; 4 Medicine, Lady Reading Hospital, Peshawar, PAK; 5 Neurology, Lady Reading Hospital, Peshawar, PAK; 6 Internal Medicine, Mardan Medical Complex Medical Teaching Institution, Mardan, PAK; 7 Internal Medicine, Ayub Medical College, Abbotabad, PAK; 8 Internal Medicine, Hayatabad Medical Complex, Peshawar, PAK; 9 Medicine, Mardan Medical Complex, Mardan, PAK

**Keywords:** child-pugh class, chronic liver disease, cutaneous manifestations, hepatitis c, pruritus

## Abstract

Background and objective: Chronic liver disease (CLD) is often accompanied by a variety of skin conditions, which may be related to the severity of the disease and to other systemic involvement. This study aimed to determine the prevalence and evaluate demographic and clinical predictors of cutaneous manifestations in patients with CLD.

Methodology: The study was a hospital-based analytical cross-sectional study conducted at the Department of Gastroenterology and Hepatology, Lady Reading Hospital (Peshawar, PAK), from February 2025 to February 2026. Non-probability consecutive sampling was used to recruit a total of 238 patients with CLD. A structured pro forma was used to collect detailed demographic, clinical, dermatological, and laboratory data. The severity of CLD was assessed using the Child-Pugh classification, and cutaneous manifestations were diagnosed through standardized clinical dermatological examination. Associations between variables were assessed using the chi-square test, and independent predictors were identified using multivariable logistic regression analysis. Variables were selected based on univariate analysis and clinical relevance.

Results: Cutaneous manifestations were present in 196 (82.35%) patients and absent in 42 (17.65%). The most common findings included pruritus in 154 (64.71%), jaundice in 147 (61.76%), xerosis in 118 (49.58%), and palmar erythema in 96 (40.34%) patients. Severity-wise, manifestations were significantly higher in Child-Pugh class C (91.78%) compared to class B (86.54%) and class A (63.93%) (p <0.001). Independent predictors of cutaneous manifestations included Child-Pugh class C (adjusted odds ratio (AOR) 3.95), hepatitis C virus infection (AOR 2.87), diabetes mellitus (AOR 2.11), male gender (AOR 1.72), and disease duration greater than five years (AOR 2.36).

Conclusion: Cutaneous manifestations are highly prevalent in patients with CLD and are significantly associated with disease severity and important clinical risk factors. These findings should be interpreted as associations rather than causal relationships. Future studies should use longitudinal multicenter designs to better establish temporal relationships and causal pathways between CLD progression and cutaneous manifestations. In clinical practice, routine dermatological examination should be incorporated into the assessment of CLD patients as a simple, low-cost tool to aid early recognition of disease severity and systemic involvement.

## Introduction

Chronic liver disease (CLD) is a significant health issue and a cause of significant morbidity and mortality around the world [1]. It covers the broad range of progressive liver diseases such as chronic viral hepatitis, alcoholic liver disease, non-alcoholic fatty liver disease, autoimmune hepatitis, and cirrhosis [2]. Repeated liver injury causes liver fibrosis, architectural changes, portal hypertension, and ultimately liver insufficiency [3]. The risk of CLD is especially significant in developing countries because hepatitis B and C infections, metabolic disorders, alcohol abuse, and delayed diagnosis of liver diseases are more common [4] in this population. Patients with CLD often have multisystem morbidities that negatively impact quality of life and health care utilization [5].

In CLD, skin is one of the most affected organs, and skin changes are one of the important clinical markers for underlying hepatic dysfunction [6]. These dermatological changes can be due to metabolic disorders, vascular alterations, hormonal dysfunction, nutritional deficiencies, immune alterations, or toxic metabolites that are produced when the liver does not function properly [7]. These include jaundice, pruritus, spider angiomas, palmar erythema, xerosis, hyperpigmentation, ecchymosis, nail changes, and caput medusa [8]. As such, some skin findings may occur relatively early in the disease and can help the clinician suspect a CLD prior to the occurrence of severe systemic complications [9].

The distribution and type of skin changes are dependent on their cause, duration, and degree of liver disease [10]. There have been several studies that have shown a higher prevalence of dermatological abnormalities with advanced liver dysfunction and cirrhosis [11]. Furthermore, demographic and clinical parameters like age, gender, diabetes mellitus, alcohol consumption, viral hepatitis status, nutritional status, and biochemical abnormalities could be factors in the incidence and/or severity of skin manifestations [12]. Although these findings are clinically relevant, dermatologic findings in CLD are not well recognized in the clinical practice setting, especially in the resource-limited healthcare environment where diagnostic tools may be limited [13].

The recognition of cutaneous signs may help in early diagnosis, early follow-up, and proper management of CLD. Knowledge of their frequency and predictors can help enhance clinical evaluation and help provide complete treatment of the patient. Hence, the present prospective cohort study was conducted to assess the frequency of the cutaneous features and their clinical predictors in patients of CLD who presented to a tertiary care hospital.

## Materials and methods

Study design and setting

This study was conducted at the Department of Gastroenterology and Hepatology, Lady Reading Hospital (Peshawar, PAK). This is a hospital-based analytical cross-sectional study aimed at determining the prevalence and clinical predictors of cutaneous manifestations in patients with CLD. Consecutive patients presenting to both inpatient and outpatient departments were screened for eligibility and enrolled after obtaining informed consent. This research was conducted between February 2025 and February 2026.

Sample size and sampling technique

A total of 238 patients with CLD were included in the study. The sample size was calculated using the sample size formula $n = \frac{Z^{2} \, p \, (1 - p)}{d^{2}}$ wher Z = 1.96 (95% confidence level), p = 0.30 (expected prevalence of cutaneous manifestations), and d = 0.06 (margin of error). By substituting the values, as shown below, the sample size was determined.

$n = \frac{(1.96)^2 \times 0.30 \times (1 - 0.30)}{(0.06)^2}$

$n = \frac{3.8416 \times 0.30 \times 0.70}{0.0036}$

$n = \frac{0.8067}{0.0036}$

$n = 224.1 \approx 224$

All eligible patients meeting the inclusion criteria during the study period were included using a non-probability consecutive sampling technique. The inclusion and exclusion criteria are shown in Table 1.

**Table 1:** Selection criteria CLD: Chronic liver disease

Inclusion criteria	Exclusion criteria
Patients aged 18 years or older	Patients with pre-existing chronic dermatological diseases unrelated to liver disease
Both male and female patients	Patients with acute liver failure
Patients with clinically diagnosed CLD	Patients with hepatocellular carcinoma (HCC)
Patients with radiologically diagnosed CLD	Patients with severe systemic diseases
Patients with laboratory-confirmed CLD	Immunocompromised patients
Duration of CLD of six months or more	Patients taking medications known to independently cause skin manifestations
Patients with CLD of any etiology	
Patients with viral hepatitis-related CLD	
Patients with alcoholic liver disease	
Patients with non-alcoholic fatty liver disease	
Patients with autoimmune hepatitis	
Patients with cryptogenic cirrhosis	

Data collection procedure

Demographic and clinical data were collected using a structured pro forma (see Appendix A) after obtaining written informed consent. Information on age, sex, cause, and duration of liver disease, presence of other chronic illnesses, drug history, alcohol consumption, and other relevant clinical symptoms was recorded. Alcohol consumption was assessed through patient self-report and categorized as current, past, or non-user based on clinical history. Drug history was reviewed to exclude medications known to independently cause cutaneous manifestations. The study subjects were thoroughly examined by trained doctors for both physical and dermatological assessment under the supervision of a consultant dermatologist.

Clinical assessment

A full clinical assessment was carried out for every patient. Severity of CLD was determined using a combination of clinical findings, laboratory investigations, and radiological imaging. The CLD diagnosis was established based on compatible clinical features (such as jaundice, ascites, and stigmata of chronic liver disease), biochemical evidence of hepatic dysfunction (abnormal liver function tests), and radiological findings on ultrasound suggestive of chronic liver disease or cirrhosis (including coarse liver echotexture, nodular liver surface, splenomegaly, or portal hypertension). Peripheral edema, hepatic encephalopathy, jaundice, ascites, hepatic decompensation, and portal hypertension were systematically documented. The Child-Pugh classification was used for staging liver disease severity where applicable.

Dermatological evaluation

A comprehensive dermatological assessment was performed under adequate lighting to identify skin changes associated with CLD. Cutaneous manifestations were defined using standard clinical dermatology criteria: pruritus (generalized itching without primary skin lesions), jaundice (yellow discoloration of skin and sclera), spider angiomas (central arterioles with radiating capillaries that blanch on pressure), palmar erythema (erythematous discoloration of the thenar and hypothenar eminences), xerosis (dry skin), hyperpigmentation (increased melanin deposition), ecchymoses (non-blanching purpuric lesions due to subcutaneous bleeding), nail abnormalities (e.g., brittle nails, leukonychia), excoriations (scratch marks due to itching), caput medusae (dilated periumbilical veins), and gynecomastia-related skin changes. Systematic documentation of all findings was performed using a standardized checklist. Cutaneous manifestations were confirmed through joint examination by a consultant dermatologist and trained physicians to ensure consistency and reduce observer bias.

Laboratory investigations

The following investigations were performed in accredited laboratory facilities: complete blood count, liver function tests, serum bilirubin, alanine aminotransferase, aspartate aminotransferase, prothrombin time, international normalized ratio, renal function tests, viral hepatitis profile, and blood glucose levels. Relevant radiological imaging reports were also reviewed to support clinical diagnosis and staging.

Study variables

The main dependent variable was the presence of cutaneous manifestations in patients with CLD. Independent variables included age, gender, duration of liver disease, etiology of CLD, Child-Pugh class, laboratory parameters, diabetes mellitus, alcohol use, and other relevant clinical characteristics.

Outcome measures

The primary outcome measure was the prevalence of cutaneous manifestations among patients with CLD. Secondary outcome measures included the frequency and pattern of specific dermatological manifestations and the identification of clinical predictors associated with cutaneous involvement.

Statistical analysis

Data were analyzed using SPSS Statistics version 26 (IBM Corp., Armonk, NY, USA). Quantitative variables such as age and duration of disease were presented as mean ± SD, while qualitative variables were expressed as frequencies and percentages. Associations between cutaneous manifestations and clinical variables were assessed using the chi-square test. Independent predictors were identified using multivariable logistic regression analysis. For regression analysis, variables were initially screened through univariate analysis, and those with p < 0.05, along with clinically relevant factors, were entered into the multivariable model.

Model performance was evaluated using the Hosmer-Lemeshow goodness-of-fit test, and multicollinearity was assessed using the variance inflation factor (VIF), with VIF < 5 considered acceptable. Adjusted odds ratios (AOR) with 95% CI were reported for significant predictors. A p-value ≤ 0.05 was considered statistically significant.

Ethical statement

The study was approved by the Institutional Review Board of Lady Reading Hospital Medical Teaching Institution (approval no. 1329/LRH/MTI) before data collection. All subjects provided their signed informed consent. All procedures were carried out in accordance with the ethical principles of the Declaration of Helsinki, and the confidentiality of patient information was carefully observed throughout the study.

## Results

A total of 238 patients with CLD were included. The baseline demographic, clinical, and laboratory characteristics of patients with CLD are shown in Table 2. The majority were aged between 46 and 60 years (89, 37.39%), followed by 31 and 45 years (71, 29.83%). Males predominated (146, 61.34%). Hepatitis C virus was the leading etiology (104, 43.70%), followed by hepatitis B virus (48, 20.17%). Diabetes mellitus was present in 97 patients (40.76%). Most patients had Child-Pugh class B disease (104, 43.70%). Clinically, portal hypertension (121, 50.84%) and ascites (112, 47.06%) were the most frequent manifestations. Mean laboratory values indicated hepatic dysfunction with elevated bilirubin (3.82 ± 2.17 mg/dL) and low albumin (2.94 ± 0.75 g/dL).

**Table 2:** Baseline demographic, clinical, and laboratory characteristics of patients with CLD (total n = 238) CLD: Chronic liver disease

Variable	Category	N (%)/Mean ± SD
Age (years)	18-30	32 (13.45%)
	31-45	71 (29.83%)
	46-60	89 (37.39%)
	>60	46 (19.33%)
Gender	Male	146 (61.34%)
	Female	92 (38.66%)
Etiology of CLD	Hepatitis C virus	104 (43.70%)
	Hepatitis B virus	48 (20.17%)
	Alcoholic liver disease	31 (13.03%)
	Non-alcoholic fatty liver disease	39 (16.39%)
	Autoimmune/cryptogenic	16 (6.72%)
Diabetes mellitus	Present	97 (40.76%)
	Absent	141 (59.24%)
Duration of CLD	<2 years	74 (31.09%)
	2 to 5 years	108 (45.38%)
	>5 years	56 (23.53%)
Child-Pugh class	Class A	61 (25.63%)
	Class B	104 (43.70%)
	Class C	73 (30.67%)
Clinical features	Ascites	112 (47.06%)
	Peripheral edema	86 (36.13%)
	Hepatic encephalopathy	39 (16.39%)
	Portal hypertension	121 (50.84%)
	Splenomegaly	97 (40.76%)
	Hepatomegaly	88 (36.97%)
Laboratory parameters	Serum bilirubin (mg/dL)	3.82 ± 2.17
	Alanine aminotransferase (ALT) (U/L)	84.61 ± 31.52
	Aspartate aminotransferase (AST) (U/L)	96.33 ± 40.21
	Serum albumin (g/dL)	2.94 ± 0.75
	International normalized ratio (INR)	1.75 ± 0.53
	Hemoglobin (g/dL)	10.82 ± 1.98
	Platelet count (×10⁹/L)	124.58 ± 46.82

**Figure 1:**
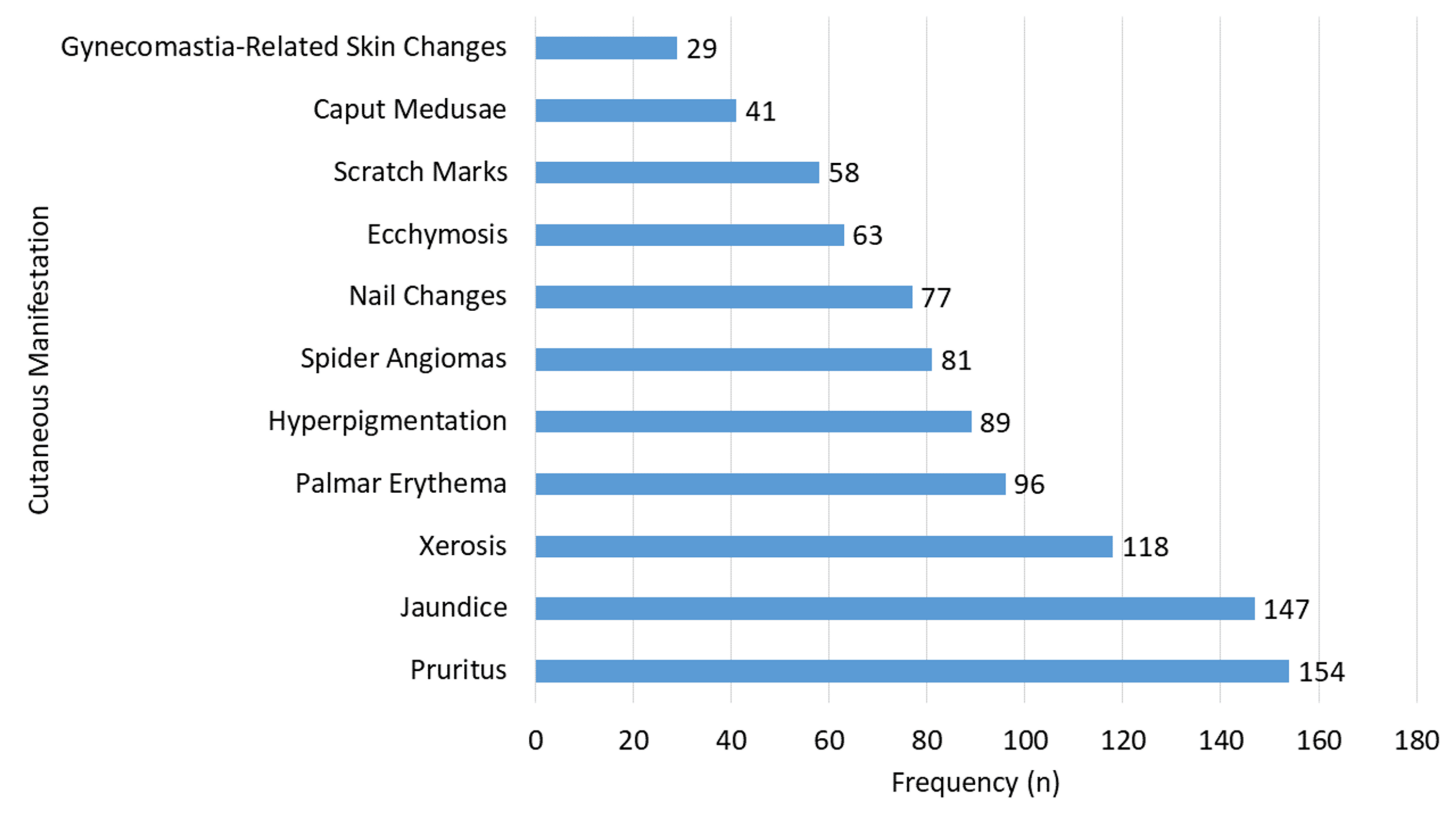
Frequency of cutaneous manifestations among patients with CLD (total n = 238) CLD: Chronic liver disease

**Figure 2:**
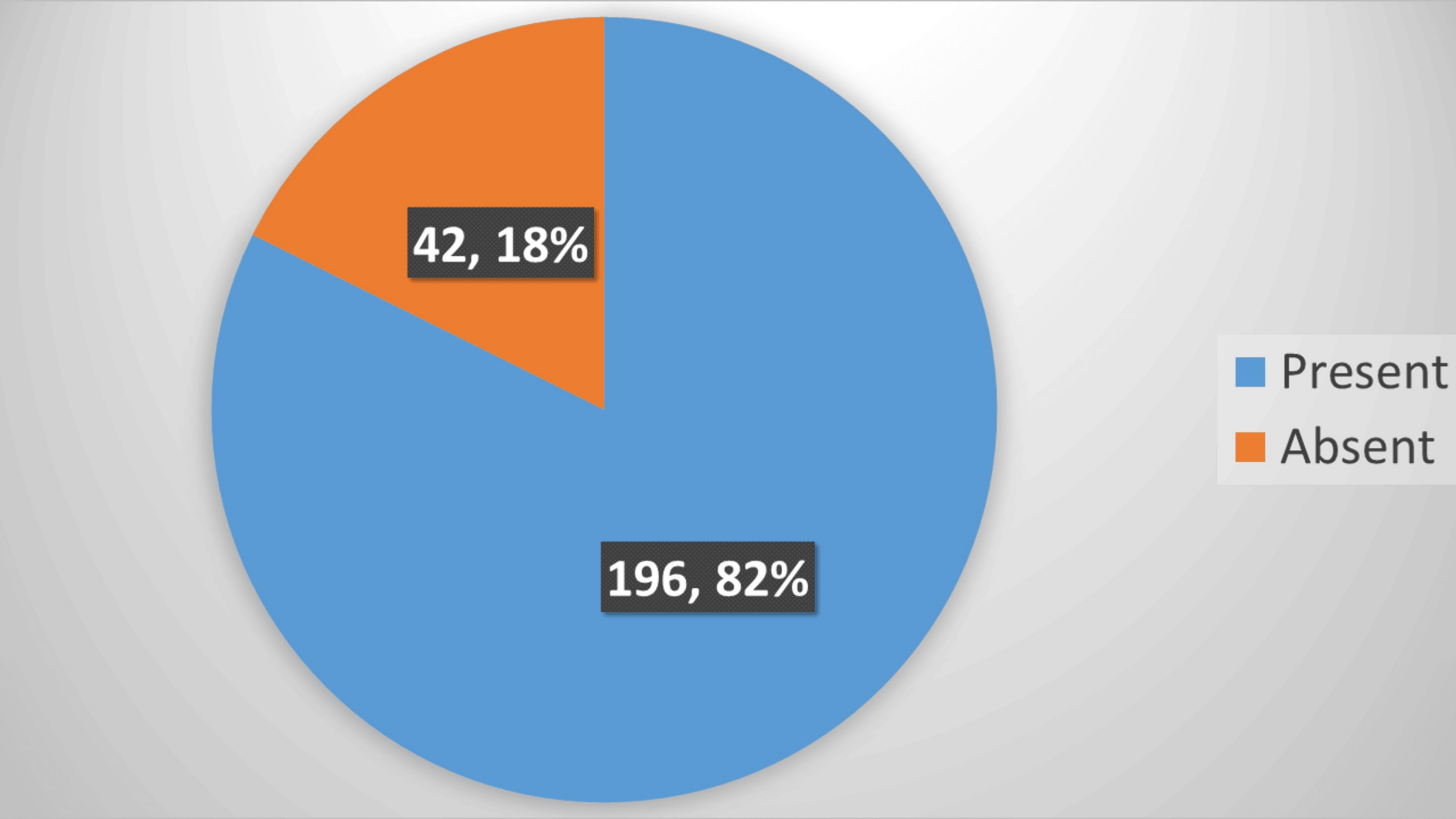
Overall frequency of cutaneous manifestations in CLD patients (total n = 238) CLD: Chronic liver disease

Pruritus was the most common cutaneous manifestation (154, 64.71%), followed by jaundice (147, 61.76%), xerosis (118, 49.58%), and palmar erythema (96, 40.34%). Less frequent findings included spider angiomas (81, 34.03%) and ecchymosis (63, 26.47%) (Figure 1). Cutaneous manifestations were present in 196 patients (82%), while 42 patients (18%) had no dermatological findings (Figure 2).

Cutaneous manifestations were significantly more common in males than females (p = 0.041), as shown in Table 3. Prevalence was highest in alcoholic liver disease and hepatitis C virus infection (p=0.002). A strong upward trend was observed with disease severity, rising from Child-Pugh class A to class C (p <0.001).

**Table 3:** Association of cutaneous manifestations with clinical variables in patients with CLD (total n = 238) CLD: Chronic liver disease

Variable	Category	Cutaneous present n (%)	Cutaneous absent n (%)	χ²	df	Cramer’s V	p-value
Gender	Male (n=146)	126 (86.30)	20 (13.70)	4.19	1	0.13	0.041
	Female (n=92)	70 (76.09)	22 (23.91)				
Etiology of CLD	Hepatitis C virus	93 (89.42)	11 (10.58)	15.89	4	0.26	0.002
	Hepatitis B virus	37 (77.08)	11 (22.92)				
	Alcoholic liver disease	28 (90.32)	3 (9.68)				
	Non-alcoholic fatty liver disease	27 (69.23)	12 (30.77)				
	Autoimmune/cryptogenic	11 (68.75)	5 (31.25)				
Child-Pugh class	Class A (n=61)	39 (63.93)	22 (36.07)	18.72	2	0.28	<0.001
	Class B (n=104)	90 (86.54)	14 (13.46)				
	Class C (n=73)	67 (91.78)	6 (8.22)				

Independent predictors of cutaneous manifestations included Child-Pugh class C (AOR 3.95), hepatitis C infection (AOR 2.87), diabetes mellitus (AOR 2.11), duration of CLD >5 years (AOR 2.36), male gender (AOR 1.72), and age >45 years (AOR 1.94), all showing statistically significant associations (Table 4).

**Table 4:** Multivariable logistic regression analysis for clinical predictors of cutaneous manifestations CLD: Chronic liver disease, AOR: Adjusted odds ratio

Variable	AOR	95% CI	p-value
Age >45 years	1.94	1.08-3.49	0.026
Male gender	1.72	1.01-2.93	0.044
Diabetes mellitus	2.11	1.19-3.76	0.010
Hepatitis C infection	2.87	1.42-5.78	0.003
Child-Pugh class C	3.95	1.88-8.31	<0.001
Duration of CLD >5 years	2.36	1.17-4.74	0.016

## Discussion

In the present study, there was a high prevalence of cutaneous manifestations among patients with CLD. This high burden highlights the clinical importance of routine dermatological assessment in CLD and is consistent with findings reported in other populations [6-8]. Pruritus and jaundice were the most frequent manifestations, followed by xerosis and palmar erythema. Similar patterns have been reported in regional studies of Pakistan, where pruritus has been identified as a leading dermatological complaint in viral hepatitis [14,15]. This consistency suggests that cutaneous involvement is a common extrahepatic feature of CLD.

In addition, vascular and pigmentary changes such as spider angiomas, hyperpigmentation, and nail abnormalities were frequently observed. These findings align with South Asian and international cohorts where such features are recognized as markers of chronic hepatic dysfunction [6,7,10]. Similar observations have been reported in regional studies, supporting the systemic vascular and hormonal effects of progressive liver disease [16,17].

A clear increase in cutaneous involvement was observed with advancing liver disease severity. This trend is consistent with international literature showing that worsening hepatic dysfunction is associated with higher dermatological burden due to metabolic, hormonal, and vascular imbalance [8,18]. Skin findings, therefore, may serve as clinical indicators of disease severity in CLD.

Etiologically, hepatitis C infection showed the strongest association with cutaneous manifestations, followed by alcoholic liver disease. Global evidence supports a strong link between hepatitis C and extrahepatic dermatological conditions through immune-mediated mechanisms [19]. Alcohol-related liver disease contributes to chronic inflammation and nutritional deficiencies, affecting skin integrity [12].

Multivariable analysis identified liver disease severity, viral hepatitis, diabetes mellitus, longer disease duration, and male gender as independent predictors of cutaneous involvement. These findings are consistent with previous studies indicating that progressive liver dysfunction and metabolic comorbidities significantly increase systemic and dermatological complications [18,20,21].

Laboratory abnormalities reflected hepatic dysfunction and have been similarly reported in the literature, where worsening liver biochemistry correlates with extrahepatic manifestations [1,2,4]. Male patients were more frequently affected than females. This gender difference has also been reported in global studies and is attributed to higher exposure to risk factors such as viral hepatitis and alcohol use among males [6,12,22].

Strengths and limitations

This study has several strengths, including its prospective observational design, relatively adequate sample size, and comprehensive evaluation of cutaneous manifestations alongside detailed clinical, laboratory, and ultrasonographic parameters. The use of standardized dermatological assessment performed by a consultant dermatologist improved diagnostic accuracy and reduced observer variability. Furthermore, the identification of independent predictors using multivariable logistic regression analysis strengthened the internal validity of the findings.

However, several limitations should be acknowledged. Being a single-center study conducted at a tertiary care hospital, the findings may be subject to referral bias and may not be fully generalizable to the broader community population. Although consecutive sampling was used, selection bias cannot be excluded due to the non-probability sampling technique. In addition, although the study is described as prospective, the analysis of associations between variables and cutaneous manifestations is essentially cross-sectional in nature, which limits the ability to infer temporal or causal relationships between risk factors and outcomes. Moreover, potential confounding factors such as nutritional status, treatment history, and detailed comorbidity severity were not fully assessed, which may have influenced the completeness of risk factor evaluation and residual confounding.

## Conclusions

Cutaneous manifestations are highly prevalent among patients with CLD, with pruritus and jaundice being the most frequently observed findings. These manifestations show a higher frequency with increasing severity of liver disease and are particularly common in patients with an advanced Child-Pugh class. Significant associations were observed with hepatitis C infection, diabetes mellitus, longer disease duration, and the male gender. These findings suggest that cutaneous manifestations are associated with the severity of CLD and may serve as useful clinical indicators of underlying hepatic dysfunction. However, given the observational nature of the study, these findings should be interpreted as associations rather than causal relationships. Recognition of these dermatological features may assist in the clinical assessment of disease severity and support timely evaluation and management of patients with CLD.

## Appendices

Appendix A

Table 5 features the structured questionnaire or pro forma used to collect demographic and clinical data for the study. 

**Table 5:** Structured questionnaire/pro forma for the assessment of demographic, clinical, laboratory, and dermatological variables in patients with CLD CLD: Chronic liver disease, ALT: Alanine aminotransferase, AST: Aspartate aminotransferase, INR: International normalized ratio, HCC: Hepatocellular carcinoma

Section	Variable/item	Response type/categories
Administrative information	Participant ID	
	Date of interview	
	Recruitment site	
	Written informed consent	Yes/no
Demographic characteristics	Age (years)	
	Age group	18-30/31-45/46-60/>60 years
	Gender	Male/female
Clinical history of CLD	Duration of CLD (years)	
	Duration category	<2 years/2-5 years/>5 years
	Etiology of CLD	Hepatitis C virus/Hepatitis B virus/alcoholic liver disease/non-alcoholic fatty liver disease/autoimmune hepatitis/cryptogenic cirrhosis
	Diabetes mellitus	Present/absent
	Other chronic illnesses	Yes/no (specify)
	Drug history	Yes/no (specify)
	Alcohol consumption history	Yes/no
Clinical assessment	Peripheral edema	Present/absent
	Hepatic encephalopathy	Present/absent
	Jaundice	Present/absent
	Ascites	Present/absent
	Portal hypertension	Present/absent
	Splenomegaly	Present/absent
	Hepatomegaly	Present/absent
	Hepatic decompensation	Present/absent
	Child-Pugh classification	Class A/B/C
Dermatological Assessment	Presence of cutaneous manifestations	Yes/no
	Pruritus	Present/absent
	Jaundice (cutaneous)	Present/absent
	Xerosis	Present/absent
	Palmar erythema	Present/absent
	Hyperpigmentation	Present/absent
	Spider angiomas	Present/absent
	Nail changes	Present/absent
	Ecchymosis	Present/absent
	Scratch marks	Present/absent
	Caput medusae	Present/absent
	Gynecomastia-related skin changes	Present/absent
	Other mucocutaneous abnormalities	Present/absent (specify)
Laboratory Investigations	Complete blood count (CBC)	Normal/abnormal
	Hemoglobin (g/dL)	
	Platelet count (×10⁹/L)	
	Serum bilirubin (mg/dL)	
	ALT (U/L)	
	AST (U/L)	
	Serum albumin (g/dL)	
	Prothrombin time (PT)	
	INR	
	Renal function tests	Normal/abnormal
	Viral hepatitis profile	HBV positive/HCV positive/negative
	Blood glucose	
Radiological assessment	Relevant imaging findings	Normal/abnormal (specify)
Outcome variables	Overall presence of cutaneous manifestations	Present/absent
	Most prominent dermatological manifestation	Specify
	Frequency of cutaneous manifestations	Percentage
Eligibility assessment	Age ≥18 years	Yes/no
	CLD duration ≥6 months	Yes/no
	Diagnosed CLD (clinical/radiological/laboratory)	Yes/no
	Pre-existing dermatological disease	Yes/no
	Acute liver failure	Yes/no
	HCC	Yes/no
	Severe systemic disease	Yes/no
	Immunocompromised state	Yes/no
	Medication causing skin manifestations	Yes/no
